# Proteomic analysis of purified coronavirus infectious bronchitis virus particles

**DOI:** 10.1186/1477-5956-8-29

**Published:** 2010-06-09

**Authors:** Qingming Kong, Chunyi Xue, Xiangpeng Ren, Chengwen Zhang, Linlin Li, Dingming Shu, Yingzuo Bi, Yongchang Cao

**Affiliations:** 1 State Key Laboratory of Biocontrol, School of Life Sciences, Sun Yat-sen University, Guangzhou, 510006, China; 2 State Key Laboratory of Livestock and Poultry Breeding, Institute of Animal Science, Guangdong Academy of Agricultural Sciences, Guangzhou 510640, China; 3 College of Animal Science, South China Agricultural University, Guangzhou, 510642, China; 4 Institute of Watershed Science and Environmental Ecology, Wenzhou Medical College, Wenzhou, 325035, China

## Abstract

**Background:**

Infectious bronchitis virus (IBV) is the coronavirus of domestic chickens causing major economic losses to the poultry industry. Because of the complexity of the IBV life cycle and the small number of viral structural proteins, important virus-host relationships likely remain to be discovered. Toward this goal, we performed two-dimensional gel electrophoresis fractionation coupled to mass spectrometry identification approaches to perform a comprehensive proteomic analysis of purified IBV particles.

**Results:**

Apart from the virus-encoded structural proteins, we detected 60 host proteins in the purified virions which can be grouped into several functional categories including intracellular trafficking proteins (20%), molecular chaperone (18%), macromolcular biosynthesis proteins (17%), cytoskeletal proteins (15%), signal transport proteins (15%), protein degradation (8%), chromosome associated proteins (2%), ribosomal proteins (2%), and other function proteins (3%). Interestingly, 21 of the total host proteins have not been reported to be present in virions of other virus families, such as major vault protein, TENP protein, ovalbumin, and scavenger receptor protein. Following identification of the host proteins by proteomic methods, the presence of 4 proteins in the purified IBV preparation was verified by western blotting and immunogold labeling detection.

**Conclusions:**

The results present the first standard proteomic profile of IBV and may facilitate the understanding of the pathogenic mechanisms.

## Background

Infectious bronchitis virus (IBV), the coronavirus of domestic chickens that causes acute, highly contagious respiratory disease, is one of the most important causes of economic loss in the poultry industry. IBV is an enveloped virus with continuous, positive and single-stranded RNA genome, which is the largest of any RNA virus characterized[1] and encodes four types of structural proteins. The spike (S) glycoprotein, together with small envelope (E) protein and matrix (M) glycoprotein, consists of the viral envelope, whereas the nucleocapsid (N) protein interacts with genomic RNA of the virus to form the viral nucleocapsid, in the invariable order 5'-S-E-M-N-3'. Proteins S, E, and M have been studied for their important roles in receptor binding and virus budding. S mediates attachment to cellular receptors and entry by fusion with cell membranes, whereas M interacting with S and N proteins is an essential component of virion and plays pivotal roles in virion assembly, budding and maturation [2,3]. In addition, S protein can inhibit host cell translation by interacting with eIF3f [4] and the interaction between M and actin facilitates virion assembly and budding [5]. E is a poorly characterized small envelope protein present in low levels in the virions. The significance of the E protein appears to be critical for viral budding. Another role for protein E is that it can promote apoptosis [6,7].

Viruses constantly adapt to and modulate the host environment during replication and propagation. To govern egress from the host cell and initiation of replication in the target cell, viruses will carry some of the host proteins when released from infected cells. Enveloped viruses particularly encoding only small proteins have the capability of incorporating numerous host proteins into or onto the newly formed viruses. It is an important prerequisite for the functional studies to know the protein composition of the purified viral particles, as it allows the analysis of specific proteins and their roles during the virus life cycle, resulting in better understanding of the infection process and the pathogenesis of viruses.

As a large number of virus complete genomes have been sequenced since 1980s, more and more host proteins in different enveloped viruses have been studied using viral proteomic approaches. Herpesviruses have been the most extensively studied in this respect, such as Kaposi's sarcoma-associated herpesvirus (KSHV) [8,9], Marek's disease virus (MDV) [10], Epstein-Barr virus (EBV) [11], human cyotomegalovirus (HCMV) [12] and murine cyotomegalovirus (MCMV) [13]. Other double-stranded DNA (dsDNA) viruses including vacciniavirus have also contributed to a better understanding of this intriguing phenomenon [14-16]. Furthermore, recent studies on identification of the incorporated host proteins in RNA viruses have also been undertaken. For retrovirus, various studies in this research area have been performed on human immunodeficiency virus type 1 (HIV-1) [17-20] and moloney murine leukemia virus (MMLV) [21]. For paramyxovirus, numerous host proteins have been found incorporated into avian influenza virus (AIV) particles and respiratory syncytial virus (RSV) particles [22-24].

To date, no study of the host proteins in the virions of coronavirus has been performed yet. In this study, we performed two-dimensional gel electrophoresis fractionation coupled to mass spectrometry identification approaches to perform a comprehensive proteomic analysis of purified IBV particles. Our analysis resulted in the identification of 2 virus-encoded structural proteins and 60 incorporated host proteins. In addition, we also discussed the functional implications of some host proteins in IBV infection and pathogenesis.

## Results

### Purification of IBV particles

**Figure 1:**
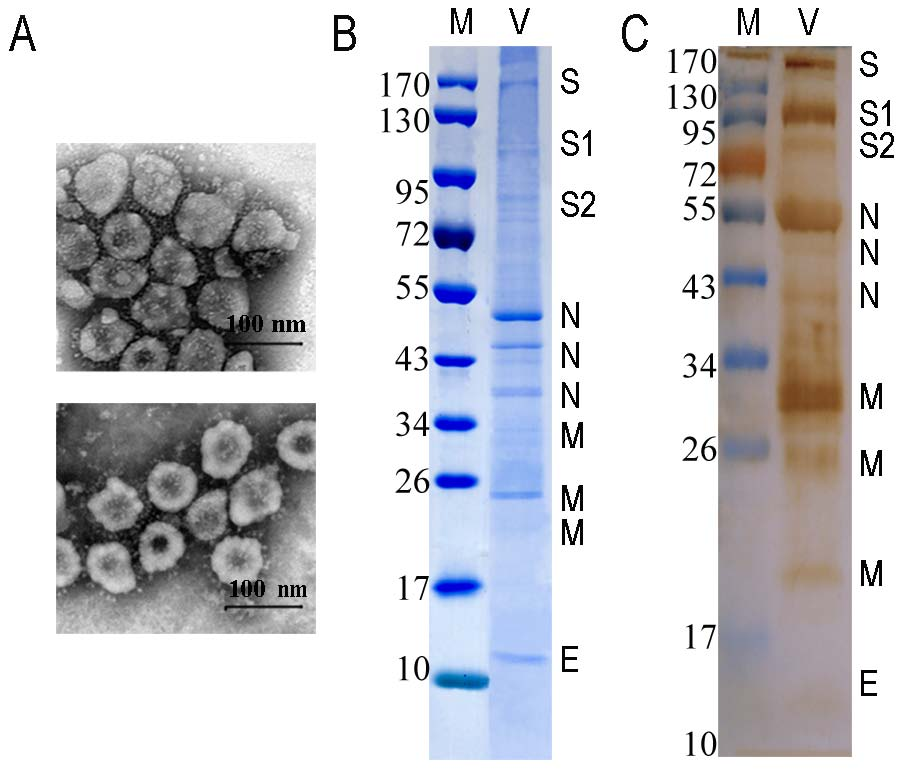
**Analysis of purified avian infectious bronchitis virus preparations**. A: specific pathogen free (SPF) chick embryo-grown major particles H52 from 30%-40% sucrose density gradients, negatively stained with 2% potassium phosphotungstate, pH 6.5. B: SDS-PAGE separation of proteins in a purified H52 preparation. 8 μg of proteins were separated on an 5-17.5% polyacrylamide gel and stained with Coomassie blue. C: Western blotting of the purified H52 virions. Viral proteins were separated on 12% polyacrylamide gel and analyzed by western blot with chicken polyclonal antibody against infectious bronchitis virus (Massachusetts). The identified viral proteins are indicated. S: spike, N: nuclecapsid, M: mebrane, E: envelope.

Viral proteomic analysis requires a highly purified preparation of virions. There was no available permissive cell line capable of supporting productive replication of IBV. Although primary chick embryo kidney cell (CEK) and chick kidney cell (CK) are capable of supporting productive replication of IBV, their poor yields prohibit them from being used for producing large quantity of IBV. In order to obtain large quantity of IBV virions, this study selected 10-day-old SPF embryonated chicken eggs for the growth of IBV strain H52. The AF with enrichment of H52 was clarified by differential centrifugation in order to remove the contamination of nuclei, mitochondria, lysosomes, peroxisomes and so on from the chicken embryo. The virus was concentrated through a 20% (wt/vol) sucrose cushion before purified over a non-linear 20%-50% sucrose-TNE (Tris-buffered saline including 50 mM Tris, 100 mM NaCl, 1 mM EDTA, pH 7.4) gradient. Two distinct types of IBV particles were isolated by sucrose density gradients. The higher density particles banded at 30%-40% sucrose-TNE gradients while the less density particles banded at 20%-30% sucrose-TNE gradients. The purity of IBV was confirmed by electron microscopy analysis following negative staining to ensure that the virions have normal viral morphology and to exclude the possible inclusions of vesicles, other cellular organelles and debris (Fig. 1A). An abundance of intact virions were observed without obvious contamination from host cellular materia. Proteins in purified virions were separated on 12% Sodium dodecylsulfate polyacrylamide gel electrophoresis (SDS-PAGE) and stained withc coomassie brilliant blue (Fig. 1B). There were also some lighter bands visible that may represent cellular proteins besides the conjectured major virus-encoded structural proteins. Furthermore, the four virus-encoded structural proteins were confirmed by immunoblotting test (Fig. 1C). Taken together, the best purification of the IBV was obtained after differential centrifugation to remove the cellular contamination and condensation through a 20% (wt/vol) sucrose cushion with a non-linear sucrose gradient.

### Proteomic analysis of purified IBV particles

**Figure 2:**
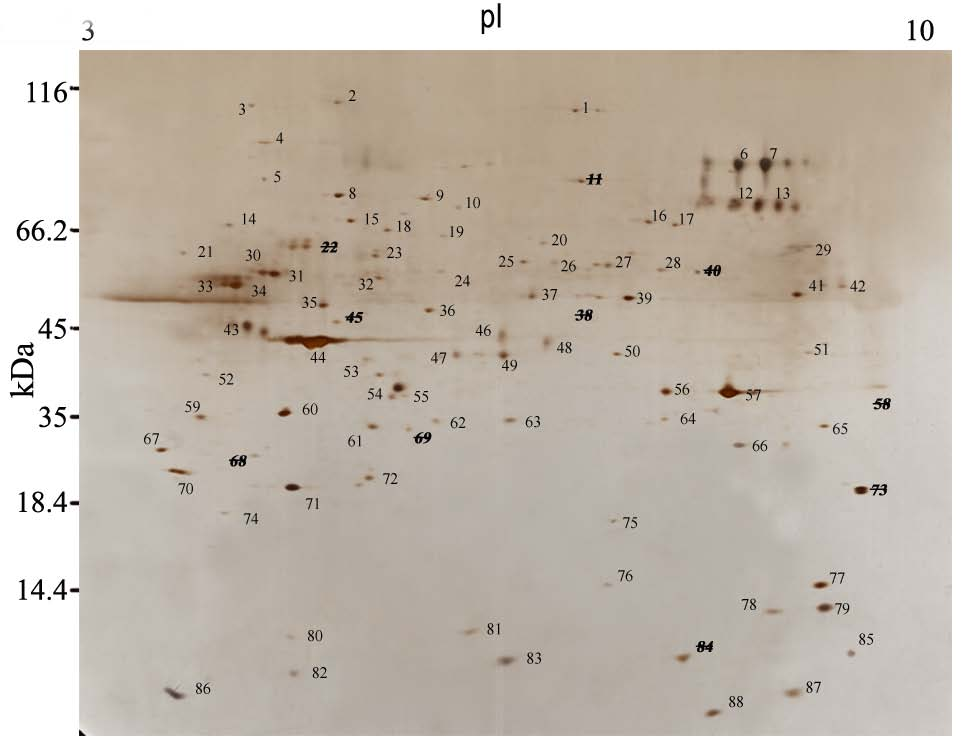
**Two-dimensional gel electrophoresis profiles of proteins in purified infectious bronchitis virus particles**. Arabic numbers indicate the protein spots; Arabic numbers with line indicate the failed identified protein spots.

To obtain a detailed protein composition profile associated with the IBV particles, the proteins in purified IBV particles were extracted for 2-DE experiments. To authenticate the results and to compensate the variability of gel electrophoresis, three independent experiments were performed with three replicate gels for each experiment. The viral protein profiles were analyzed by 2-D with 250 μg of protein. After the electrophoresis separation, gels were stained with silver and processed for image analysis. For IBV particle-associated proteins separated in the pH3-10 range, 88 protein spots were detected (Fig. 2).

### Identification and classification of IBV-associated proteins

To identify the proteins associated with IBV particles, all protein spots detected in the gels were excised and in-gel digested with trypsin followed by MALDI-TOF/TOF (Matrix-assisted laser desorption/ionizationtime of flight mass spectrometry) analysis. Database search analysis revealed that 2 virus-encoded structural proteins and 60 host proteins were successfully identified. Detailed information of the full set of the identified proteins is listed in Table 1; additional file 1.

**Figure 3:**
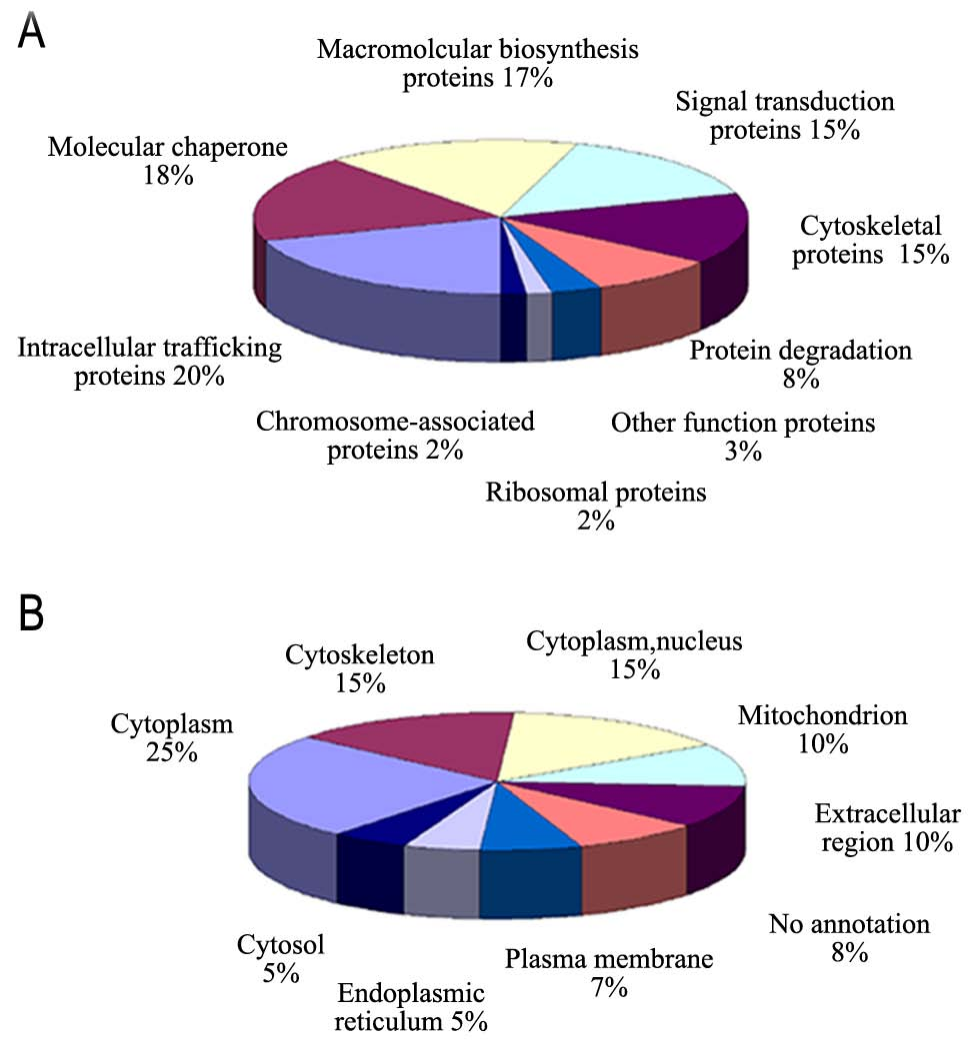
**Subcellular location and functional category of the identified host proteins**. (A) Subcellular location; (B) Functional category.

To better understand the host proteins incorporated with IBV virion and their roles played in IBV infection, these proteins were categorized with biological processes according to Uniprot Knowledge database (Swiss-Prof/TrEMBL) and Gene Ontology Database. The identified 60 host proteins were comprised of cytoskeleton proteins, molecular chaperone, macromolecular biosynthesis proteins, signal transport proteins and glycolytic enzymes (Table S1; additional file 1). These host proteins were located mainly in the cytoplasm, including cytoskeleton, cytosol and mitochondrion (Fig. 3).

### Confirmation of cellular protein incorporated into IBV particles

**Figure 4:**
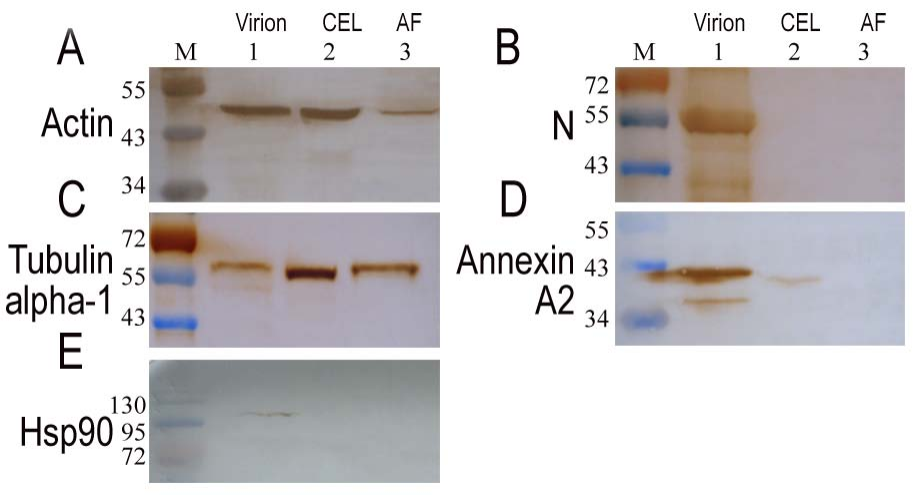
**Confirmation of host proteins incorporated into infectious bronchitis virus particles with western blotting**. 8 μg of purified virions from allantoic fluid (AF) (lane 1) and 15 μg of proteins extracted either from the normal 10-day-old specific pathogen free (SPF) chick embryo(CEL)(lane 2) or from the AF of the uninfected 10-day-old specific pathogen free (SPF) embryonated chicken eggs which were parallelly subjected to our standard density centrifugation procedure (lane 3) were subjected to western blot analysis with antibodies against the following proteins: (A) Actin, (B) Nucleocapsid (N) protein of the IBV, (C) Tubulin alpha-1, (D) Annexin A2, (E) Hsp90. Numbers to the left are molecular weight markers (M).

To confirm the presence of host proteins in the purified IBV particles after the identification of them by proteomic method, we performed immunoblotting experiments. IBV preparation purified from AF was analyzed for the presence of N protein, actin, HSP90, annexin A2 and tubulin (Fig. 4). Extracts from 10-day-old specific pathogen free (SPF) embryonated chicken eggs were included as a positive control. When analyzing the results of virion proteomic studies, the challenge is to prove that the host proteins are really an part of the virion and that they are not just attached non-specifically to the outside of the virus. To address this question, the AF from uninfected 10-day-old SPF embryonated chicken eggs were parallelly subjected to our standard density centrifugation procedure and the protein extracts from 30%-40% sucrose gradient was used as negative control. Gradient-purified virions and the control were separated on 12% SDS-PAGE gels, transferred to PVDF membranes, and probed with the appropriate antibodies. As shown in Fig. 4, actin, tubulin and annexin A2 were both found in the purified virions and positive control but not in the AF extracts from uninfected 10-day-old SPF embryonated chicken eggs. HSP90 is a member of the heat shock protein family which is upregulated in response to stress and has low abundance in unstressed cells. In present study, we detected it only in the purified virions but not in normal cells. It is an expectable result that we also detected actin and tubulin in the AF extracts from uninfected 10-day-old SPF embryonated chicken eggs which resulted from their high concentrations in all eukaryotic cells and subcellular fractions.

**Figure 5:**
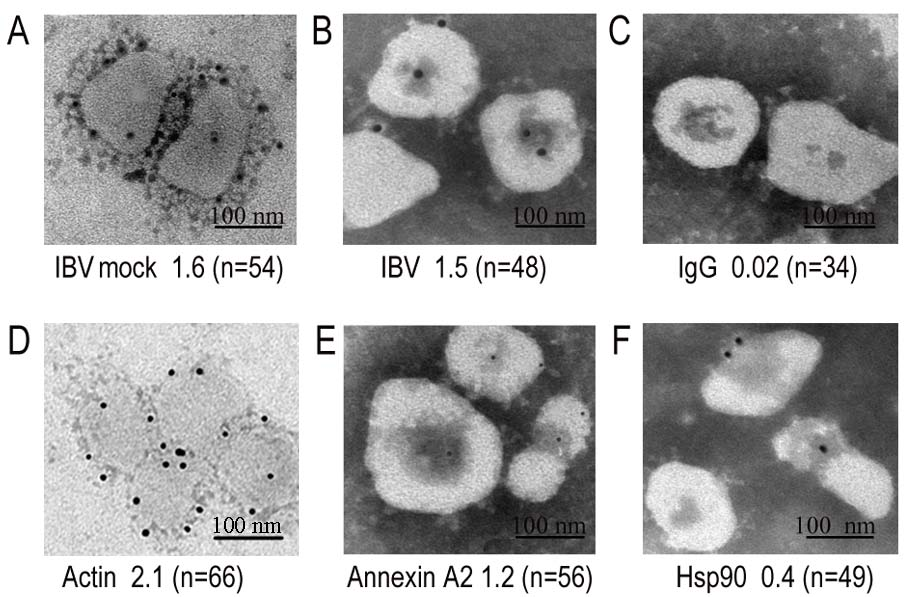
**Immunogold labeling of host proteins in purified infectious bronchitis virus (IBV) particles**. Purified IBV particles were either mock treated (A) or subjected to digestion with bromelain (B-F) followed by concentration through a sucrose cushion and then immunogold labeled with antibodies against (A) IBV mock, (B) IBV, (C) normal mouse IgG, (D) Actin, (E) Annexin A2, (F) Hsp90. Labeled virions were fixed in 2% paraformaldehyde for 5 min at RT and treated with Triton X-100 (0.2%) in PBS (pH 7.4) for 5 min and then blocked with 5% BSA in PBS-Tween 20 (pH 7.4) for 30 min at RT. After being incubated with primary and secondary antibody, the virions were negatively stained with 2% sodium phosphotungstate and visualized by electron microscopy (30,000 × magnification). Arabic numbers shown outside the blanket indicate the quantity of gold particles per virion while numbers shown inside the blanket indicate the quantity of virions counted.

To provide additional evidence that the host proteins are not just derived from a microvesicle or exosome that co-purified with the virus, we used the bromelain protease protection assay which has been shown to efficiently remove microvesicles from IBV virion preparations[25]. Protease treatment of the purified virus preparation strips proteins off any contaminating microvesicles and off the outside of virus particles, such as S protein. In doing so, the microvesicles become lighter than the virions and therefore the virions can be isolated by density centrifugation. Proteins that are inside the virion are protected by the lipid envelope and therefore will remain after the protease treatment. And then immunogold labeling of the purified virions was performed (Fig. 5). Virions were either mock treated or subjected to digestion with bromelain and then were incubated with antibodies against actin, annexin A2, HSP90, IBV Massachusetts strain and secondary gold antibodies followed by negative staining. One or two gold particles located on the surface of a virion could be seen for HSP90. This was significantly less compared with the degree of other labelings which is consistent with the fact that there is most likely far more actin, annexin A2 present on the virions than HSP90. In addition, the abundance of actin detected in the 2-DE gels is much higher than that of HSP90.

## Discussion

Virus exploits multiple host proteins during infection for successful entry, replication, egress, and evasion. This is especially true for RNA viruses because they encode only little proteins. Learning the protein composition profile of the infectious viral particle is prerequisite for studying the role of host proteins during infection. To our knowledge, incorporation of host proteins in the enveloped-virus family *Coronaviridae *has not been investigated so far. In this study, we revealed the presence of virus-encoded proteins in infectious bronchitis particles and for the first time confirmed the incorporation of host proteins. A total of 2 viral and 60 host proteins associated with purified IBV particles were identified.

In the present study, we failed to obtain M and E protein while other two structural proteins N and S were identified successfully. N protein is easy to identify because it is the most abundant virus-derived protein produced throughout the process of the virus infection, whereas S protein as a major structural protein of IBV located on the surface of viral particles is also easy for identification because it is large (about 175 kDa) and has many tryptic cleavage peak in the MS analysis. The identification of M and E protein of coronavirus by MS has been thought to be a difficult task due to their properties, especially in the case of E protein for the following reasons [26]. First, E protein is a very hydrophobic protein. Second, it is low-abundant in the virions. Third, E is a low molecular weight protein with the mass about 12 kDa. Fourth, E protein contains two cysteines, which may form disulfide bonds within itself or with other proteins and make E protein difficult to be reduced and subsequently digested.

Of the total host proteins, 39 have also been described to be present in virions of quite diverse virus families, such as herpesviruses, poxviruses, paramyxovirus and retroviruses [27,28]. There are some explanations that the incorporated host proteins are common to other virus families. First, they are all enveloped viruses. Enveloped viruses contain the viral genome and core proteins wrapped within one or more membranes which are acquired from the host cell during virus assembly and budding. These viruses all share some fundamental feature in the particular stage of their life-cycle and these host proteins are involved in the common process. Second, these host proteins would be either highly abundant cytosolic proteins or enriched at the virus budding sites. Several of the highly abundant cytosolic proteins found within both IBV and other viral particles are beta actin, tubulin, annexins and enolase. Proteins enriched at the virus budding sites including HSP70, HSP90 and GAPDH are also identified in IBV and other viruses [9,11,12,14,19,24].

Some host proteins may be specially incorporated into the virions. In this study, 21 of the total host proteins are reported for the first time. The identified host protein functions in diverse biological processes and some functional groups are analyzed. These proteins participate in a broad array of cellular functions and are involved in many processes in the viral life cycle. The potential roles of some of these proteins are discussed below in relation with IBV infection, pathogenesis and early host antiviral response.

Numerous viral proteins interact with cytoskeletal elements. Many viruses, such as retroviruses, herpesviruses and picornaviruses, even contain the main cytoskeletal element actins in their infectious particles. The transport machinery of actins are proven to be critical at almost every step along the infectious cycle [29]. Actin has been found in preparations from several types of retroviruses and paramyxoviruses. For coronavirus, an association of M with cytoskeletal elements has been reported [5], which indicates an essential function of actin in the replication cycle of coronavirus IBV. In our studies, actin and tubulin were all present in the interior of infectious bronchitis particles and this observation most likely reflects their active participation in moving the viral components to assembly sites. Actin and tubulin have been characterized as the major folding substrates for CCT (Chaperonin containing TCP-1, also termed TRiC). Both cytoskeletal proteins require in vivo and in vitro the interaction with CCT to fold to their native states [30]. CCT is the most different and complicated protein of all group II chaperonins in eukaryotic cytosolic chaperonins, which might be involved in the assistance of the folding of a small set of proteins. In addition to the already mentioned actin and tubulin, CCT has been found to interact either in vitro or in vivo with other cytoskeletal proteins, cell division control protein 20, protein phosphatase type 2A, and guanine nucleotide-binding protein (G protein) beta subunit [31], which are all found to be associated with infectious bronchitis particles in present study. It's pleasantly surprising to find that certain viral proteins such as the Epstein Barr virus-encoded nuclear protein (EBNA-3), the hepatitis B virus capsid and the type D retrovirus Gag polyprotein are also folded by CCT [32-34]. Thus, CCT may have an important role in infectious bronchitis viral proteins assembly.

Other cytoskeletal proteins found to be associated with infectious bronchitis particles are actin-related proteins, WD repeat containing protein, destrin and annexin. Several annexin family members (A2, A5 and A11) were identified in purified infectious bronchitis particles. Annexins are a well-known multigene family of Ca^2+ ^regulated phospholipid-binding and membrane binding proteins with diverse functions. The presence of annexin A2 is thought to support viral binding, fusion and replication [35-39]. Annexin A5, which interacts with annexin A2, has the opposite effect by preventing fusion, which possibly indicates a potential regulatory role [38]. Annexin A2 tightly binds to a member of the S100 family of calcium-binding proteins, S100A10 (p11). Upon binding, annexin A2 and p11 form a heterotetramer which is capable of binding two membrane surfaces simultaneously, which potentially promotes fusion events and also plays a role in exocytosis [40]. The p11 protein was also detected by our analysis, suggesting that IBV is also incorporated this complex. Other S100 family members such as S100A6 and S100A11 were also detected in viral samples and could play various roles in fusion and membrane organization [41,42].

Heat-shock proteins (HSPs) have been known as multifunctional proteins. They facilitate the folding and unfolding of proteins, participate in vesicular transport processes, prevent protein aggregation in the densely packed cytosol and are involved in signaling processes. Most, but not all, HSPs are molecular chaperones. Several viruses require host molecular chaperones for entry, replication, and assembly, as well as other steps in viral production [43,44]. HSP70 and HSP90 have been found incorporated into IBV. HSP70 interacts with various viral proteins and may be involved in the assembly of adenovirus [45], enterovirus [46], vaccinia virus [47] and hantaan virus [48]. Alternatively, upon entry into susceptible target cells, virion-associated HSP70 might participate in early events of infection. For example, HSP70 might actively uncoat the viral capsid in a manner similar to its role in the uncoating of clathrin cages [49]. HSP70 and HSP90 have been shown to interact with hepatitis B virus reverse transcriptase and to facilitate the initiation of viral DNA synthesis from hepatitis B virus pregenomic RNA [50,51]. For sendai virus (SV), the viral proteins synethsis will be inhibited as long as HSP70 synthesis occurs [52]. Thus, HSP70 in IBV virions might serve a similar function in the virus life cycle. The chaperone HSP90 has been identified as an essential factor in the folding and maturation of picornavirus capsid proteins [53]. The involvement of HSP90 in viral replication has also been reported for many viruses and it has been demonstrated that HSP90 inhibition blocks viral replication [54]. Recently, a role for HSP90 in the control of hepatitis C, flock house and influenza virus polymerase function has been shown [55-61] and it has been proposed that HSP90 is a major host factor that is of central importance for viral replication for a wide spectrum of RNA viruses [56], which implies the crucial roles of HSP90 in IBV replication. The importance of HSP90 for the replication of multiple viruses opens up an interesting possibility for developing new antiviral therapies which have not yielded drug-resistant viruses [62].

Some proteins involved in the glycolytic pathway were identified, such as aldehyde dehydrogenase 9 family, member A1 (ALDH9A1), glyceraldehyde-3-phosphate dehydrogenase (GAPDH), alpha-enolase, which were identified in other viral particles, like HIV-1, MMLV, HCMV, KSHV and AIV [8,12,19,21,24]. Some studies have suggested that several glycolytic enzymes interact with microtubules and tubulin [63-65] and may also contribute to transcription of RNA virus genomes. In higher eukaryotes, enolase is found as a dimer of subunits, α, β, or γ. All enolase isoforms from mammalian have been reported that are capable of stimulating transcription of SVgenome [66]. GAPDH is a well-characterized key enzyme in glycolysis, but recent evidence suggests it also has RNA binding properties and binds to the untranslated RNA sequences of several different viruses, including human parainfluenza virus type 3(hPIV3), Japanese encephalitis virus (JEV), hepatitis A virus (HAV) and hepatitis B virus (HBV) as well as hepatitis C virus (HCV) [67-71]. In the case of hPIV3, GAPDH has been reported to inhibit actin-dependent in vitro transcription and is also present in purified virions [67,72]. In vitro data indicates that GAPDH serves a negative regulatory role in hPIV3 transcription and in a phosphorylation-dependent on manner [72].

In addition to these host proteins associated with enveloped viruses, the roles of which in the virus life cycles have been studied well, we also identified 21 host proteins in purified infectious bronchitis particles, which have not been described to be present in other virions of quite diverse virus families, such as apolipoprotein A-I (apoA-I), fatty acid-bingding protein 3, ovalbumin, TENP protein, tumor protein translationally controlled-1, transthyretin and so on. ApoA-I, a major constituent of high-density lipoproteins, alters plasma membrane morphology by participating in the reverse transport of cholesterol binding with ATP-binding cassette transporter A1 [73], and activates the small GTP-binding protein cdc42-associated signaling including apoA-I induced cholesterol efflux, protein kinases, and actin polymerization [74]. What important is that apo A-I can inhibit herpes simplex virus (HSV)-induced cell fusion at physiological concentrations. This function may be related to the structure of apoA-I and before long its amphipathic peptide analogue was also found to inhibit cell fusion, both in HIV-1-infected T cells and in recombinant vaccinia-virus-infected CD4+ HeLa cells expressing HIV envelope protein on their surfaces [75]. The results indicate that amphipathic helices may be useful in designing novel antiviral agents that inhibit penetration and spreading of enveloped viruses. Ovalbumin is the main protein found in egg white, making up 60-65% of the total protein. The chicken ovalbumin upstream promoter transcription factors (COUP-TFs), members of the steroid/thyroid hormone receptor superfamily, binds to a negative regulatory region in the human immunodeficiency virus type 1 long terminal repeat (LTR). LTR contains a negative regulatory element which downregulates the rate of LTR-directed transcription and HIV-1 replication [76]. The interaction between ovalbumin and NP from influenza A virus as well as glycoprotein C from the herpes simplex type I virus was reported long time ago [76]. The TENP protein from *G. gallus*, however, was isolated as a transiently expressed gene in neural precursor cells in retina and brain, and has been proposed to function in the transition to cell differentiation in neurogenesis. After expressed in chicken embryonic fibroblast cells, TENP was immunodetected in membrane fractions, implying that TENP might be a membrane protein as predicted by a computer analysis of its primary sequence [77]. To date, there have been no reports about TENP associated with virus, but it's an enriched and abundant protein identified in purified infectious bronchitis particles which suggests to us that it may be a requisite host protein in IBV life cycles.

## Conclusions

The present study 1) provides the first proteomic analysis of infectious bronchitis particles, 2) establishes the most comprehensive proteomic index of IBV and 3) shows that most of the virion incorporated host proteins have central roles in virus life cycle. Although some proteins may be associated with virus biology, further investigation of the function of these host proteins may facilitate the understanding of the pathogenic mechanisms.

## Methods

### Propagation and purification of IBV

The IBV strain H52 was obtained from Qianyuanhao Biological Corporation Limited (Beijing, China). Virus was propagated in 10-day-old specific pathogen free (SPF) embryonated chicken eggs (Beijing Merial Vital Laboratory Animal Technology Co, Ltd, Beijing, China) for 48 h at 37°C. The allantoic fluid (AF) with enrichment of IBV H52 was clarified by differential centrifugation. AF was first centrifugated at 3,000 × g for 30 min and then the supernatant was centrifugated at 12,000 × g for 30 min. Clarification and all subsequent centrifugations were performed at 4°C. The virus was sedimented through 5.5 ml of 20% (wt/vol) sucrose in TNE buffer (50 mM Tris, 100 mM NaCl, 1 mM EDTA, pH 7.4) by centrifugation in a 70Ti rotor (Beckman Coulter, Optima™ L-100XP Preparative ultracentrifuge) at 75,000 × g for 1.5 h. Condensed Virions were then diluted with 1.0 ml TNE buffer and centrifuged to equilibrium in 11.5 ml non-linear 20%-50% sucrose-TNE gradients at 75,000 × g for 2.5 h in a SW41 rotor (Beckman Coulter, Optima™ L-100XP Preparative ultracentrifuge). Purified virions were diluted with TNE buffer and pelleted by sedimentation at 75,000 × g for 1.5 h in a SW41 rotor to remove the sucrose. The purified IBV pellets were stored at -80°C until use.

### Sample preparation

The purified IBV particles were dissolved in about 300 μl lysis buffer (7 M urea, 2 M thiourea, 2% Triton X-100, 65 mM DTT, 2% Biolyte pH 3-10) and incubated for 60 min at 4°C. Then the lysis solution was sonicated for 4 min (pulse durations of 2 s on and 3 s off) in an ice bath sonicator. The viral protein samples were prepared when the indiscerptible sediments were wiped off by centrifugation at 12,000 × g for 30 min. The supernatant was collected and the concentration of the prepared protein samples was determined by the Bio-Rad protein assay kit II according to the manufacturer's instructions. The samples were then aliquoted and stored at -80°C until used for further analysis.

### Protease treatment of virions

Purified virus particles treated with bromelain (BB0243, BBI) at 0.2 mg/ml in 50 mM DTT (pH 7.2) in Dulbecco's phosphate buffered saline (PBS) at 37°C for 15 min. After incubation, the treated virus was directly centrifuged to equilibrium in 11.5 ml non-linear 20%-50% sucrose-TNE gradients at 75,000 × g for 2.5 h in a SW41 rotor (Beckman Coulter, Optima™ L-100XP Preparative ultracentrifuge). Purified virions were diluted with TNE buffer and pelleted by sedimentation at 75,000 × g for 1.5 h in a SW41 rotor to remove the sucrose and then subjected to immunogold labeling and electron microscopy analysis.

### Two-dimentional gel electrophoresis (2-DE) and silver staining

Two-dimentional gel electrophoresis analysis was performed using 18 cm immobile DryStrip (IPG strips, pH 3-10 non-linear, GE Healthcare). First, 100 μl samples containing 250 μg protein were added into 400 μl sample rehydration buffer (7 M urea, 2 M thiourea, 2% (w/v) CHAPS, 65 mM DTT, 0.2% Bio-lyte pH 3-10) and incubated for 30 min at 37°C prior to their separation by isoelectric focusing (IEF) in the first dimension. The IPG strips were rehydrated at 20°C for 12 h by a passive rehydration method. IEF was carried out for a total of 45 kvh at 20°C on an Ettan IPGphor III electrophoresis unit (GE Healthcare). Second, IPG strips were further transferred onto the second dimension of gel electrophoresis. Before this step the IPG strips were reduced and alkylated in a equilibration buffer containing 50 mM Tris-HCl, pH 8.6, 6 M urea, 2% SDS and 30% glycerol supplemented with 1% (w/v) DL-Dithiothreitol (DTT) or 2.5% iodoacetamide (IAA) instead of DTT for 15 min. Subsequently, the viral protein samples were separated at 140 V on linear 5%-17.5% Sodium dodecyl sulfate gradient polyacrylamide gel (SDS-PAGE) in Tris: glycine buffer (192 mM glycine, 25 mM Tris, 0.1% SDS, pH 8.3) for about 10 h. Third, proteins in the gel were stained by the modified silver staining method compatible with MS [78] and the gels were scanned at a resolution of 600 dpi using ImageScanner™ III (GE Healthcare).

### Tryptic digestion and MALDI-TOF/TOF analysis of peptide

Gel pieces (1.0 mm^3^) containing the whole protein spots from the 2D gel were cut and washed three times with 50 mM carbonic acid, monoammonium salt (NH_4_HCO_3_, Amresco). These gel pieces were destained with 15 mM potassium ferricyanide (K_3_Fe(CN)_6_, Amresco) and 50 mM sodium thiosulfate (NaS_2_O_3_, Amresco) in 50 mM NH_4_HCO_3 _and dehydrated in 100% acetonitrile (ACN, Wako) until gel pieces turn to white. After dring in SpeedVac concentrator (Thermo Savant, USA) for about 100 min, gel pieces were incubated with 12.5 ng/μl trypsin (Sequenceing grade, Promega) to cover dry gel pieces completely at 37°C overnight. The gel pieces were then extracted three times in 50% ACN water solution containing 5% trifluoroacetic acid (TFA, Wako). The supernatant was pooled and dried thoroughly in SpeedVac. Protein digestion extracts were resuspended with 5 μl of 0.1% TFA and then the peptide samples were mixed (1:1) with a matrix consisting of a saturated solution of α-cyano-4-hydroxycinnamic acid (α-CCA, Sigma) in 50% ACN containing 0.1% TFA. 0.8 μl aliquot was spotted onto stainless steel target plates. Peptide mass spectra were obtained on an Applied Biosystem/MDS SCIEX 4800 MALDI TOF/TOF plus mass spectrometer. Data were acquired in positive MS reflector using a CalMix5 standard to calibrate the instrument (ABI4800 Calibration Mixture). Mass spectra were obtained from each sample spot by accumulation of 900 laser shots in an 800-3500 mass range. For MS/MS spectra, the 5-10 most abundant precursor ions per sample were selected for subsequent fragmentation and 1200 laser shots were accumulated per precursor ion. Both the MS and MS/MS data were interpreted and processed by GPS Explorer software (V3.6, Applied Biosystems), then those obtained MS and MS/MS spectra per spot were combined and submitted to MASCOT search engine (V2.1, Matrix Science, London, U.K.) by GPS Explorer software and searched with the following parameters: trypsin as the digestion enzyme, one missed cleavage site, partial modification of cysteine carboamidomethylated and methionine oxidized, none fixed modifications, MS tolerance of 60 ppm, MS/MS tolerance of 0.25 Da. MASCOT protein score in IPI_CHICKEN (V3.49) database (based on combined MS and MS/MS spectra) of greater than 57 (p ≤ 0.05) or in NCBInr database of greater than 67 (p ≤ 0.05) was accepted.

### Western blotting analysis

Mouse monoclonal antibodies against actin (MAB1501) and HSP90 (05-594) were purchased from millipore. Rabbit polyclonal antibodies against Annexin A2 (ab40943) and Tubulin alpha-1 (ab4074), and chicken polyclonal antibody against IBV (Massachusetts) (ab31671) were purchased from Abcam. Mouse monoclonal antibody against nucleoprotein of IBV (3BN1) was purchased from HyTest Ltd. For control, the AF from 10-day-old SPF embryonated chicken egg performed with the same protocol as the purification of IBV particles and the protein extracted from the normal 10-day-old SPF embryonated chicken eggs included for western blot analysis. Samples were separated at 120 V on linear 5%-17.5% SDS-PAGE with 5% stacking gels in Tris: glycine buffer for about 3 h. For purified virus, 10 μg of total proteins were used per lane. For the control, a total of 15 μg proteins were loaded. After separated by SDS-PAGE, the proteins were transferred to a polyvinylidene fluoride membrane (PVDF, P/N 66485, BioTrace, Pall Corporation). The membrane was blocked in freshly prepared 5% bovine serum albumin (BSA) with 0.05% Tween-20 for 2 h at room temperature with constant agitation. The PVDF membrane was washed three times with Tris buffered saline plus 0.2% Tween 20 (TBST) and then incubated with properly diluted primary antibodies for 2 h at room temperature or overnight with agitation at 4°C. Anti-rabbit or anti-mouse immunoglobulin G antibody conjugated to horseradish peroxidase (HRP) (00001-14, Proteintech Group, Inc) was used as the secondary antibody and the PVDF membrane was incubated in it for 1 h at room temperature. The chemiluminescence system (AR1022, Boster Bio-Technology Co. LTD) was used for detection of antibody-antigen complexes.

### Immunogold labeling and electron microscopy

Rabbit polyclonal antibody against chicken IgG (15 nm Gold) (ab41500), goat polyclonal against rabbit IgG (5 nm Gold) (ab27235) and goat polyclonal against mouse IgG (10 nm Gold) (ab27241) were purchased from Abcam. Purified IBV particles were suspended in PBS (pH 7.4) and then were collected onto 230-mesh formwar-coated nickel grids and adsorbed on the grids for 5 min. The viruses were fixed in 2% paraformaldehyde for 5 min at RT and treated with Triton X-100 (0.2%) in PBS (pH 7.4) for 5 min and then blocked with 5% BSA in PBS-Tween 20 (pH 7.4) for 30 min at RT. All grids were then blocked with blocking buffer (5% BSA, 5% normal serum, 0.1% cold water skin gelatin, 10 mM phosphate buffer, 150 mM NaCl, pH 7.4) for 30 min. After washing with PBS, immobilized virions were incubated for 1.5 h with 50 μg/ml primary antibody (in 1% BSA), and washed three times for 5 min in PBS/1% BSA. Anti-rabbit or anti-mouse immunoglobulin G coupled to 10 nm colloidal gold particles was used as the secondary antibody and virions were incubated in it for 40 min at room temperature. The grids were then washed extensively with PBS, washed twice more with distilled water to remove excess salt and negatively stained with 2% sodium phosphotungstate for 1 min. Negatively stained virions were examined on a scan and transmission electron microscope.

## Abbreviations

2D: two-dimensional; 2-DE: two-dimensional electrophoresis; SDS-PAGE: Sodium dodecylsulfate polyacrylamide gel electrophoresis; MS: mass spectrometry; MALDI-TOF: Matrix-assisted laser desorption/ionization time of flight mass spectrometry; SPF: specific pathogen free; AF: allantoic fluid; BSA: bovine serum albumin; DTT: dithiothreitol; IAA: iodoacetamide; ACN: acetonitrile; TFA: trifluoroacetic acid; α-CCA: α-cyano-4-hydroxycinnamic acid; TNE: Tris-buffered saline including 50 mM Tris; 100 mM NaCl; 1 mM EDTA: pH 7.4; PBS: Phosphate-buffered saline; TBS: Tris-Buffered Saline; TBST: Tris buffered saline plus 0.2% Tween 20; HRP: horseradish peroxidase; pI: isoelectric Point; MW: molecular weight.

## Competing interests

The authors declare that they have no competing interests.

## Authors' contributions

QK performed the main proteomic experiments and data analysis and drafted the manuscript. CX created the detailed experimental design. XR and CZ contributed to the initial phase of the proteomic experiments. LL and DS assisted in the propagation and purification of IBV. YB and YC helped conceive the research. All authors read and approved the final manuscript.

## Supplementary Material

Additional file 1 **Table S1**. Host proteins in purified infectious bronchitis particles identified by 2DE-MS/MS. Click here for file
