# Supplementary material for: Proteomic analysis of purified coronavirus infectious bronchitis virus particles
Source: Proteome Sci. 2010 Jun 9;8:29. doi: 10.1186/1477-5956-8-29 (PMC2909931; doi:10.1186/1477-5956-8-29)
Supplement: Additional file 1 — Table S1. Host proteins in purified infectious bronchitis particles identified by 2DE-MS/MS. [file 1477-5956-8-29-S1.DOC]

Table S1. Host proteins in purified infectious bronchitis particles identified by 2DE-MS/MS

| N0.^a^ | Protein Name | Subcellular location^b^ | Accession no.^c^ | Protein Score^d^ | *MW*(kDa) and *pI*^e^ | Pep. no.^f^ | Reported in other viruses |
| --- | --- | --- | --- | --- | --- | --- | --- |
| 1 | programmed cell death 6 interacting protein | C | IPI00573261 | 146 | 97.25/6.03 | 11 | HCMV[12] |
| 2 | major vault protein | C, R | IPI00597028 | 573 | 93.77/5.32 | 41 | HCMV[12] |
| 3 | heat shock protein 90kDa beta (Grp94), member 1 | ER | IPI00570770 | 117 | 91.50/4.83 | 14 | HIV[19] |
| 4 | heat shock 90kDa protein 1, alpha | CY | IPI00596586 | 90 | 84.01/5.01 | 10 |  |
| 5 | heat shock 70kDa protein 5 | ER,CS | IPI00590375 | 213 | 71.97/5.12 | 16 | HIV[19], AIV[23] |
| 6、7 | spike glycoprotein S1 | M, V | gi|57791727 | 76 | 58.87/7.77 | 6 | SARS[26] |
| 8 | heat shock 70kDa protein 8 | C | IPI00818933 | 399 | 70.96/5.43 | 18 | RSV[22],MMLV[21], MCMV[13], HIV[18],VV[14-16] |
| 9 | heat shock 70 kDa protein | M,CS | IPI00582091 | 130 | 69.71/5.53 | 9 | HIV[19], AIV[24] |
| 10 | similar to scavenger-receptor protein | M | IPI00810955 | 60 | 62.39/4.87 | 15 | AIV[24],HCMV[12],HIV[19], VV[14], KSHV[9] |
| 12、13 | spike glycoprotein S2 | M, V | gi|148533261 | 83 | 69.33/5.48 | 5 | SARS[26] |
| 14 | protein phosphatase 2, regulatory subunit A, beta isoform | PP | IPI00811766 | 58 | 6.50/4.77 | 1 | HIV[19], MCMV[13], HCMV[12] |
| 15 | similar to vacuolar proton-ATPase A-subunit, partial | C | IPI00590769 | 71 | 28.45/5.58 | 6 |  |
| 16 | WD repeat domain 1 | CYT | IPI00581678 | 145 | 66.52/6.19 | 17 |  |
| 17 | chaperonin containing TCP1, subunit 6A (zeta 1) | C,CCT | IPI00592971 | 315 | 57.77/6.36 | 16 | HIV[19], EBV[11] |
| 18 | chaperonin containing TCP1, subunit 5 (epsilon) | C,CCT | IPI00575509 | 262 | 59.68/5.53 | 18 | HIV[18,19], VV[14,15], EBV[11], MCMV[13],RSV[22], |
|  |  |  |  |  |  |  | KSHV[8,9],AIV[23,24], MMLV[21], HCMV[12], |
| 19 | t-complex 1 | C,NH,MIC,CCT,P | IPI00584300 | 359 | 60.99/5.43 | 22 | HIV[19] |
| 20 | chaperonin containing TCP1, subunit 7 | C,CCT | IPI00580968 | 212 | 60.31/5.96 | 14 | HIV[19], EBV[11] |
| 21 | protein disulfide isomerase precursor | C | IPI00596673 | 237 | 57.37/4.69 | 12 | EBV[11] |
| 23 | chaperonin containing TCP1, subunit 8 (theta) | C,CCT | IPI00575397 | 189 | 59.62/5.41 | 16 | EBV[23] |
| 24 | protein disulfide-isomerase A3 precursor | ER | IPI00575271 | 342 | 56.15/5.76 | 25 | EBV[23] |
| 25 | chaperonin containing TCP1, subunit 2 (beta) | C,CCT | IPI00579548 | 159 | 57.40/5.89 | 12 | EBV[11] |
| 26、27、28 | similar to aldehyde dehydrogenase 9 family, member A1 | C,N | IPI00585063 | 166 | 56.09/7.81 | 11 |  |
| 29 | nucleocapsid protein | C,G, V | gi|12484114 | 105 | 45.12/9.64 | 14 | SARS[26] |
| 30、32、82 | tubulin, alpha 1c | MIC,PC | IPI00822685 | 272 | 49.81/4.99 | 12 | HIV[19], AIV[23,24], VV[14,16], EBV[11], HCMV[12], KSHV[8] |
| 31 | tubulin, alpha 2 | MIC,PC | IPI00585941 | 251 | 50.03/4.94 | 13 | AIV[24],HCMV[12], KSHV[8,27] |
| 33 | tubulin, beta 2c | MIC,PC | IPI00837495 | 297 | 49.70/4.79 | 15 |  |
| 34 | tubulin, beta 2 | MIC,PC | IPI00591483 | 287 | 49.64/4.78 | 14 |  |
| 35 | rab-GDP dissociation inhibitor 2 | PM,C | IPI00681909 | 200 | 50.68/5.73 | 19 | HIV[19,20],MMLV[21], AIV[24], HCMV[12], KSHV[9] |
| 36 | ARP3 actin-related protein 3 homolog | CYT | IPI00587398 | 360 | 47.39/5.62 | 18 |  |
| 37、46、 | TENP protein | M | IPI00598229 | 566 | 47.40/5.57 | 8 |  |
| 47、48、49、55 |  |  |  |  |  |  |  |
| 39 | enolase 1 | C,PHC | IPI00575584 | 90 | 47.28/6.17 | 8 | HIV[17,19,20], VV[15], EBV[11], HCMV[12], KSHV[8,9] |
| 41 | annexin A11 | C,NE,NUC,ME | IPI00823286 | 446 | 53.12/7.66 | 22 |  |
| 42 | fibrinogen beta chain | EXR,FC | IPI00588322 | 685 | 54.55/7.84 | 32 | VV[16] |
| 43 | ovalbumin | EXR | IPI00583974 | 575 | 42.85/5.19 | 13 | HIV[17,19],VV[14],EBV[11], HCMV[12], KSHV[8,9] |
| 44、72 | actin, gamma 1 propeptide | CYT | IPI00837485 | 640 | 41.77/5.31 | 26 | HIV[17,19],VV[14],EBV[11], HCMV[12], KSHV[8,9] |
| 50 | ARP1 actin-related protein 1 homolog A, centractin alpha | CYT | IPI00595766 | 259 | 42.62/6.19 | 12 |  |
| 51 | glyceraldehyde-3-phosphate dehydrogenase | MI | IPI00822919 | 142 | 43.69/8.81 | 9 |  |
| 52 | 40S ribosomal protein SA | C,NEU | IPI00590239 | 262 | 33.00/4.8 | 8 |  |
| 53 | 26S proteasome subunit, non-ATPase, 13 | CY, PC | IPI00601716 | 380 | 42.72/5.37 | 16 |  |
| 54 | beta actin | CYT | IPI00655503 | 400 | 41.71/5.29 | 19 |  |
| 56、57、65 | annexin A2 | EXT,BM,S | IPI00577039 | 325 | 38.62/6.92 | 12 | HIV[19] |
| 59 | MBprotein 4b | C | IPI00582041 | 266 | 25.14/4.73 | 9 |  |
| 60 | similar to annexin VIII |  | IPI00585409 | 560 | 36.71/5.24 | 23 | HIV[19] |
| 61 | guanine nucleotide binding protein, subunit beta-2-like 1 | PM | IPI00822306 | 349 | 37.28/5.75 | 12 |  |
| 62 | annexin A5 | C | IPI00820196 | 430 | 36.19/5.61 | 20 | HIV[19], HCMV[12] |
| 63 | similar to nucleoside diphosphate kinase DR-nm23 |  | IPI00581010 | 59 | 15.89/9.67 | 1 | HIV[19] |
|  |  |  |  |  |  |  |  |
| 64 | 32 kDa protein | MBV | IPI00586374 | 209 | 31.74/6.55 | 7 | HIV[19], AIV[24] |
| 66 | syntenin | C,N,PM | IPI00583507 | 561 | 32.04/7.01 | 13 |  |
| 67 | 14-3-3 protein epsilon | M | IPI00579092 | 343 | 29.16/4.63 | 18 |  |
| 70 | 14-3-3 protein zeta | M | IPI00578632 | 410 | 27.76/4.73 | 18 |  |
| 71 | apolipoprotein A-I | EXT, HDL | IPI00580765 | 536 | 30.66/5.58 | 22 |  |
| 74 | tumor protein, translationally-controlled 1 | EXT,MB | IPI00821768 | 276 | 19.52/4.9 | 8 |  |
| 75 | similar to adhesion molecule with Ig-like domain 2 | C,N | IPI00597314 | 64 | 58.42/8.44 | 4 |  |
| 76 | cell division cycle 42 | PM ,C | IPI00818072 | 479 | 21.42/6.15 | 8 | HIV[19] |
| 77 | destrin | C, CYT | IPI00602594 | 403 | 18.52/7.52 | 14 | EBV[11] |
| 78、79 | LOC776282 hypothetical protein, partial |  | IPI00813608 | 167 | 9.07/8.56 | 4 |  |
| 80 | transthyretin | EXT | IPI00820372 | 791 | 14.97/4.98 | 7 |  |
| 81 | fatty acid-binding protein 3 | CY | IPI00583669 | 82 | 14.81/5.92 | 8 | HIV[19], AIV[23,24], VV[14,16], EBV[11], HCMV[12], KSHV[8] |
| 83 | S100 calcium binding protein A11 | C,N,RU | IPI00599279 | 166 | 11.41/6.08 | 6 | MMLV[21],KSHV[8] AIV[23,24], VV[14,16],EBV[11], HCMV[12], |
| 85 | hemoglobin subunit alpha-A | M, CY | IPI00575926 | 171 | 15.42/8.54 | 7 | MMLV[21],KSHV[8] AIV[23,24], VV[14,16],EBV[11], HCMV[12], |
| 86 | S100 calcium binding protein A6 | C,N,NE,RU | IPI00572547 | 237 | 10.27/4.91 | 3 |  |
| 87 | S100 calcium binding protein A10 | MI | IPI00595453 | 184 | 11.28/6.82 | 7 | HIV[18-21], AIV[23,24], VV[14,16] |
| 88 | ubiquitin C | C,N | IPI00820812 | 315 | 17.62/8.12 | 5 | AIV[24] |

^a^ Protein numbers positioned on 2D gel.

^b^ The locations of the protein are indicated as follows: BM, basement membrane; C, cytoplasm; CCT, chaperonin-containing T-complex; CS, cell surface; CY, cytosol; CYT, cytoskeleton; ER, endoplasmic reticulum; EXT, extracellular region; FC, fibrinogen complex; G, Golgi; HDL, high-density lipoprotein particle; M, membrane; MB, multivesicular body; MBV, Cic membrane-bounded vesicle; ME, melanosome; MI, mitochondrion; MIC, microtubule; N, nucleus; NE, nuclear envelope; NEU, nuclear euchromatin; NH, nuclear heterochromatin; NUC, nucleoplasm; P, pericentriolar material; PC, protein complex; PHC, phosphopyruvate hydratase complex; PM, plasma membrane; PP, protein phosphatase type 2A complex; R, ribonucleoprotein complex; RU, ruffle; S, sarcolemma; V, virion

^c^ Accession numbers for international protein index and NCBI database (accessible at <http://www.ebi.ac.uk/IPI/IPIhelp.html>; <http://www.ncbi.nlm.nih.gov/>).

^d^ MASCOT protein score in IPI_CHICKEN (V3.49) database (based on combined MS and MS/MS spectra) of greater than 57 (*p≤0.05*) or in NCBInr database of greater than 67 (*p≤0.05*) were accepted.

^e^ Theoretical molecular mass and *pI*.

^f^ Number of ploypeptides detected from this protein by MS/MS.
